# Design and Synthesis of Immunoconjugates and Development of an Indirect ELISA for Rapid Detection of 3, 5-Dinitrosalicyclic Acid Hydrazide

**DOI:** 10.3390/molecules13092238

**Published:** 2008-09-23

**Authors:** Yu-Dong Shen, Shi-Wei Zhang, Hong-Tao Lei, Hong Wang, Zhi-Li Xiao, Yue-Ming Jiang, Yuan-Ming Sun

**Affiliations:** 1 College of Food Science, South China Agricultural University, Guangzhou 510642, P. R. China; E-mails: sydscau@yahoo.cn (Y-D. S), zsw_8506@163.com (S-W. Z), hongtao@scau.edu.cn (H-T. W); gzwhongd@163.com (H. W.); scauxzl@scau.edu.cn (Z-L. X); 2 South China Botanical Garden, Chinese Academy of Sciences, Guangzhou 510650, P. R. China

**Keywords:** Nifursol, DNSH, Hapten design, Indirect ELISA

## Abstract

In this study novel immunoconjugates were designed, synthesized and then used to develop a rapid, specific and sensitive indirect ELISA method to directly detect residues of 3,5-dinitrosalicyclic acid hydrazide (DNSH), a toxic metabolite of nifursol present in chicken tissues. The hapten DNSHA was first designed and used to covalently couple to BSA to form an immunogen which was immunized to rabbits to produce a polyclonal antibody against DNSH. Furthermore, a novel 3,5-dinitrosalicylic acid-ovalbumin (DNSA-OVA) immunoconjugate structurally different from DNSHA-OVA was designed and used as a “substructural coating antigen” to improve the sensitivity of an indirect ELISA analysis for a direct DNSH detection. Based on the “substructural coating antigen” concept, an optimized indirect ELISA method was established that exhibited good specificity and high sensitivity for detecting DNSH, with a cross-reactivity of less than 0.1% (excluding the parent compound nifursol), IC_50_ of 0.217 nmol/mL and detection limit of 0.018 nmol/mL. Finally, a simple and efficient analysis of DNSH samples in chicken tissues showed that the average recovery rate of the indirect ELISA analysis was 82.3%, with the average coefficient of variation 15.9%. Thus, the developed indirect ELISA method exhibited the potential for a rapid detection of DNSH residues in tissue.

## Introduction

**Figure 1:**
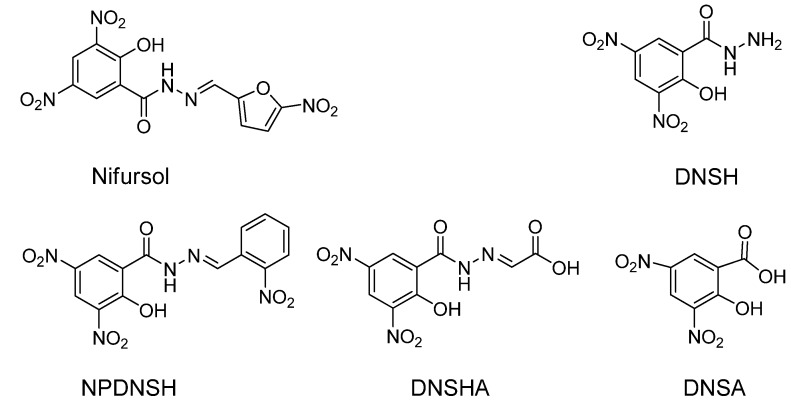
The structures of nifursol, DNSH, NPDNSH, DNSA and DNSHA [2,3,4].

Nifursol is a member of the nitrofuran antibiotics family (Figure 1), which is used extensively as a feed additive for the prevention of histomoniasis. Due to the potentially harmful effects of nifursol on human health, its use in food-producing animals has been banned in the European Union since 2002 [1]. As nifursol was rapidly metabolized to form the metabolic marker 3,5-dinitrosalicyclic acid hydrazide (DNSH, Figure 1) which can persist for a long time *in vivo*, several laboratories have focused on development of DNSH detection methods [2,3,4]. At present, the detection of DNSH is mainly at the μg/kg level by HPLC-MS methods, which are based on the analysis of the DNSH derivative 2-hydroxy-3,5-dinitro-*N*'-(2-nitrobenzylidene)benzohydrazide (NPDNSH, Figure 1) [2,3,4]. Disadvantageously, these analytical methods not only require a time-consuming and troublesome derivatization step and expensive apparatus but also have low sample throughput. 

Immunoassay is an efficient screening technique for monitoring of illegal and harmful chemicals in food and the environment, which exhibits the great advantages of speed and high sample throughput [5]. To the best of our knowledge, there have been no reports on the development of a specific antibody to DNSH and an immunoassay for a direct detection of DNSH. This study reports for the first tme the synthesis of immunoconjugates for DNSH, the preparation of a polyclonal antibody against DNSH and the development of an indirect competitive enzyme-linked immunosorbent assay (indirect ELISA) for rapidly analyzing DNSH residues present in chicken tissue. At the same time, this study developed a “substructural coating hapten” strategy to improve the precision of indirect ELISA for detecting DNSH.

## Results and Discussion

### Immunogen design and synthesis

**Figure 2:**
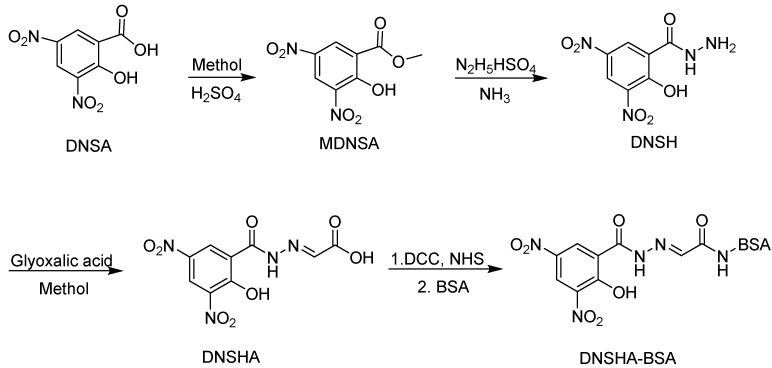
Synthesis of immunogen DNSHA-BSA [10,11,12,13].

The structural design of the hapten is an important step in the development of immunoassays for small organic analytes. As the structure of the hapten used to form an immunogen can remarkably affect the sensitivity and specificity of the antibody, the hapten should be a near perfect mimic of the structure of the target analyte [6,7,8]. On the other hand, as organic molecules of less than 1,000 Da possess no antigenicity, they must be covalently conjugated to a carrier protein by introducing a linker with an active terminal group on its organic molecular structure to form a complete antigen to smoothly elicit a specific immune response. Furthermore, the arm used to couple to a carrier protein was another key role in hapten design, which should not elicit antibody recognition in itself [8]. Generally, an appropriate linear hydrocarbon handle is necessary. Our previous studies showed that a short unsaturated linear chain which was attached to target molecule can remarkably improve the capability to evoke a specific immune response [9]. In this study, a short unsaturated arm based on glyoxalic acid was directly introduced into the target analyte to form a DNSHA hapten which was covalently linked to the carrier protein bovine serum albumin (BSA) to produce a DNSHA-BSA immunogen (Figure 2).

NMR and MS analyses, as indicated by in the Experimental, confirmed that the DNSHA hapten was prepared. The DNSHA-BSA immunogen was synthesized by the active ester method [13]. UV spectra showed qualitative differences between the carrier protein and conjugate in the maximum absorbance of hapten, which suggested the presence of DNSHA-BSA. Furthermore, the ratio of DNSHA coupling to BSA was determined to be 21:1 by the TNBS method [14].

### Design, synthesis and choice of coating antigens

The structure of the hapten used to form a coating antigen has a direct effect on the immunoassay [15]. Generally, immunohapten and target analyte have a similar structure and their affinities for an antibody are almost identical. Thus, homologous coating using an immuno-hapten-OVA conjugate as a coating antigen usually results in a similar or weaker recognition of the antibody towards the target analyte. The “substructural coating hapten”, a major structure of which was one of the fragments of target analyte structure, can improve remarkably the competitive recognition sensitivity of antibody to a target analyte, which could result from less interaction regions or points of the “substructural coating hapten” with the pocket area of the antibody than those of the analyte. Based on this concept, DNSA was designed as a “substructural coating hapten” for detecting DNSH by indirect ELISA. In addition, in order to prove the validity of the “substructural coating hapten” concept, standard curves of DNSH were established under optimum conditions using DNSA-OVA as coating antigen and DNSHA-OVA as a control, respectively. As we had expected, the result shows that the IC_50_ and detection limit of the indirect ELISA with DNSA-OVA as coating antigen were much better than those of the indirect ELISA with DNSHA-OVA (Table 1), which suggested that the “substructural coating hapten” strategy for indirect ELISA improvement was rational and efficient.

**Figure 3:**
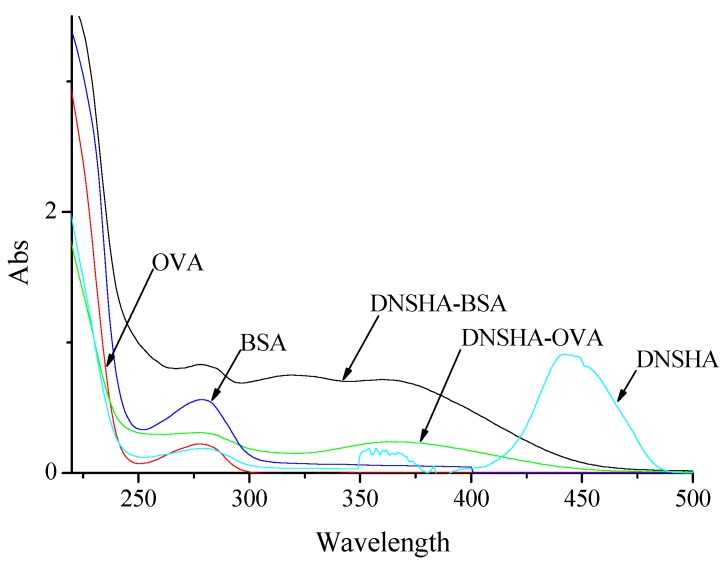
UV spectra of BSA, OVA, DNSHA-BSA, DNSHA-OVA and DNSHA.

**Figure 4:**
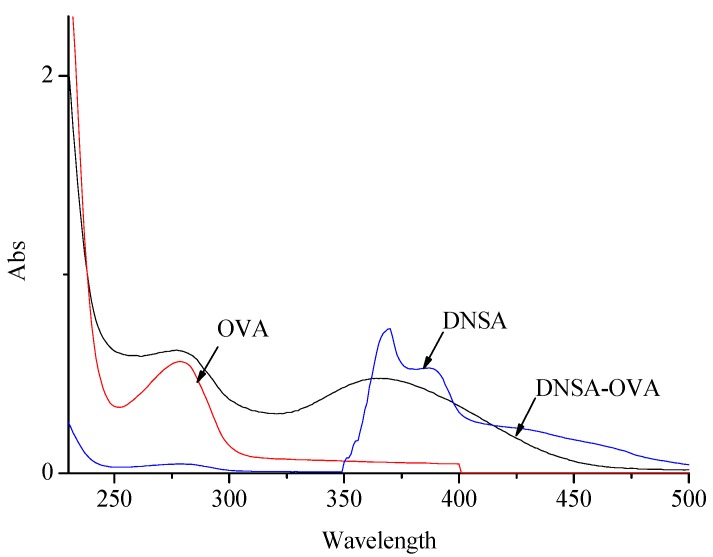
UV spectra of OVA, DNSA-OVA and DNSA.

Coating antigens were also synthesized by the active ester method with OVA instead of BSA [13]. UV spectra show qualitative differences between OVA and conjugates (DNSHA-OVA or DNSA-OVA) in the maximum absorbance of corresponding haptens (DNSHA or DNSA), as shown in Figure 3 and Figure 4, which suggested the presence of DNSHA-OVA and DNSA-OVA. The molar ratio of DNSHA and DNSA coupling to OVA were determined to be 11:1 by the TNBS method [14].

**Table 1:** Indirect ELISA evaluation for DNSH with different coating antigens.

Immunogen	Coating antigen	IC_50_ (nmol/mL)	Detection limit (nmol/mL)
DNSHA-BSA	DNSHA-OVA	10.328	0.892
	DNSA-OVA	0.217	0.018

**Table 2:** Cross-reactivity of the antibody with various chemicals.

Competitor	IC_50_ (nmol/mL)	Cross-reactivity (%)
3,5-Dinitrosalicyclic acid hydrazide (DNSH)	0.217	100
Nifursol	0.069	314.5
3-Amino-2-oxazolidinone (AOZ)	>1000	<0.1
5-Methylamorfolino-3-amino-2-oxazolidone (AMOZ)	>1000	<0.1
1-Aminohydantoin (AHD)	>1000	<0.1
Semicarbazide (SEM)	>1000	<0.1
Ciprofloxacin	>1000	<0.1
Enrofloxacin	>1000	<0.1
Clenbuterol	>1000	<0.1
Salbutamol	>1000	<0.1
Ractopamine	>1000	<0.1
Malachite green	>1000	<0.1

### Evaluations of antibody specificity and indirect ELISA

**Figure 5:**
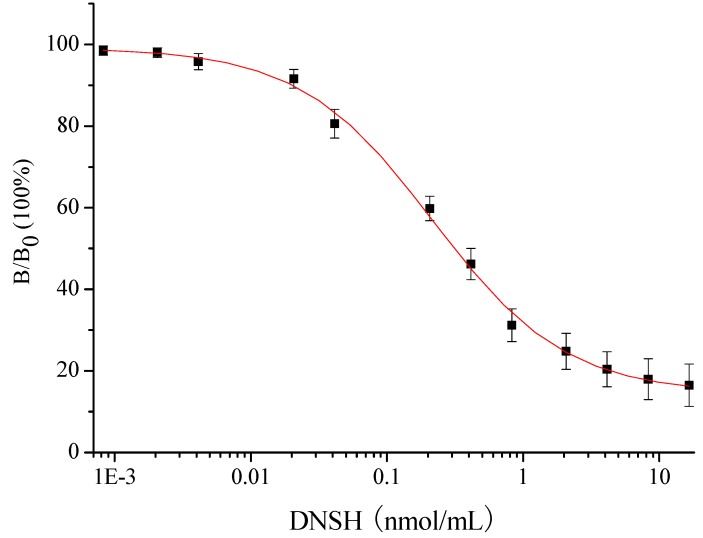
Standard curve of indirect ELISA for DNSH (n=3).

A DNSH standard curve (Figure 5) was prepared using DNSA-OVA as coating antigen, with a coating concentration of 1000 ng/mL, antibody dilution of 1:2000, competitive time of 1 h, sheep anti-rabbit IgG dilution of 1:10000 and reaction time of 20 min. The IC_50_ (concentration causing 50% inhibition of binding) was 0.217 nmol/mL. The limit of detection (the concentration causing 90% inhibition of binding) was 0.018 nmol/mL. The linear detection range (the concentration correspondong to 20% to 80% inhibition of binding) was 0.046 to 1.029 nmol/mL. In addition, under the optimized indirect ELISA conditions, the specificity of the antibody was estimated using DNSA-OVA as coating antigen and ten frequently-used chemicals as competitors (Table 2). The results show that the cross-reactivity rates for the above-mentioned ten competitors, excluding nifursol (the parent compound of the analyte), were all less than 0.1%, which suggested the antibody possessed a high specificity for detection of DNSH and the parent compound nifursol (Table 2). However, nifursol could be quite rapidly metabolized into DNSH *in vivo*, so its high cross-reactivity rate didn’t affect the indirect ELISA’s application to DNSH in real samples. The study indicated that the indirect ELISA method could be practical for development of commercial ELISA kits for monitoring DNSH residues in chicken tissues.

### Sample analysis

*Pretreatment of samples.* Generally, the DNSH present is bonded with animal tissues *in vivo* and it appears in a free form by acid hydrolysis [4]. The chicken tissues mixed with DNSH were extracted with water. Ether was used to degrease the samples because of insolubility of DNSH in ether, while methanol was used to remove tissue proteins from aqueous solution with DNSH. A pretreatment period of less than 50 min was used in this study.

*Analysis of samples.* The indirect ELISA method was used to detect DNSH present in chicken samples. The analysis results are summarized in Table 3. All coefficients of variation were below 18.4% and the average recovery was 82.3%, which suggested that good precision was obtained in this study. 

**Table 3:** Analysis of DNSH present in chicken tissues by indirect ELISA (n=3).

FortificationLevel (nmol/mL)	Mean ± S.D.(nmol/mL)	Recovery(%)	CV(%)
0.10	0.076 ± 0.014	76.0	18.4
0.20	0.17 ± 0.03	85.0	17.6
0.80	0.69 ± 0.09	86.2	13.0
2.00	1.64 ± 0.24	82.0	14.6
Means		82.3	15.9

## Conclusions

Based on the concept of “substructural coating antigen”, an indirect ELISA for direct DNSH detection, with an IC_50_ value of 0.217 nmol/mL and a limit of detection of 0.018 nmol/mL, was developed in this study. The indirect ELISA was applied for DNSH determination in chicken samples, with a pretreatment and detection period of less than 2.5 h. Coefficients of variation were about 18.4% and the mean recoveries were 82.3%. Thus, this indirect ELISA with high sensitivity and good stability could potentially be used to develop commercial ELISA kits for a sensitive and rapid detection of DNSH residues.

## Experimental

### Chemicals and reagents

3,5-Dinitrosalicylic acid (DNSA), *N*-hydroxysuccinimide (NHS), dicyclohexylcarbodiimide (DCC), 3,3’,5,5’-tetramethylbenzidine (TMB), bovine serum albumin (BSA) and ovalbumin (OVA) were purchased from Sigma Chemical Company. *N,N*-Dimethylformamide (DMF), Tween-20 and methanol were obtained from Damao Co. Ltd (Tian Jin, P.R. China). Thin-layer chromatography (TLC) was done using 0.25-mm precoated silica gel GF254 on aluminum sheets purchased from Qingdao Haiyang Chemical Co. Ltd. (Qingdao, P.R. China). New Zealand white rabbits were supplied by the Guangdong Medical Experimental Animal Centre. Enzyme immunoassay grade horseradish peroxidase (HRP), peroxidase-labeled goat antirabbit IgG were obtained from Wuhan Boster Biotech Co. The other chemicals were purchased from Alfa Corporation. Polystyrene ELISA plates were obtained from Guangzhou Jiete Biotech Co. and then treated with a Multiskan MK2 microplate washer (Thermo Labsystems). Absorbance in ELISA analysis was recorded with a Multiskan MK3 microplate reader (Thermo Labsystems). HPLC-MS analyses were performed using an Agilent HP1100 series (Agilent, Palo Alto, CA). Ultraviolet-visible (UV) spectra were obtained on a UV-160A Shimadzu spectrophotometer (Kyoto, Japan) and ^1^H-NMR spectra were recorded using a DRX-600 NMR spectrometer (Bruker, Germany-Switzerland). The buffers used in this study were as follows: 0.1 mol/L phosphate buffer saline (PBS) containing 2 mol/L NaOH for sample neutralization, 50 mmol/L carbonate buffer (pH 9.6) for coating, 10 mmol/ L phosphate buffer saline (PBS) with 0.1% Tween-20 for washing, 0.1 mol/L sodium acetate (pH 5.5) for substrate buffer and 2 mol/L H_2_SO_4_ as the stopping reagent. In addition, the substrate solution for horseradish peroxidase was prepared by addition of 10 mL of above-mentioned PBS substrate buffer and 150 μL of 1% (w/v) TMB solution into DMF and 2.5 μL of 6% (w/v) H_2_O_2__._

### Synthesis of methyl 3,5-dinitrosalicylate (MDNSA)

DNSA (2.28 g) was dissolved in methanol (50 mL), and the solution was heated under reflux with stirring for 24 h after the addition of 98% H_2_SO_4_ (0.5 mL). About half of the solution was removed under vacuum before Na_2_CO_3_ was added until no gas bubbles were observed. The obtained residue was dissolved in water (100 mL) and MDNSH (2.1 g) was separated by filtration and washed 3 times with water. APCI-MS analysis (negative) *m/z* 241 [M-H]^- ^; ^1^H-NMR (600 MHz, CDCl_3_ and TMS): δ 9.03 (d, *J*=3.0 Hz, 1H, ArH), 9.01 (d, *J=*3.0 Hz, 1H, ArH) and 4.12 (s, 3H, CH_3_).

### Synthesis of 3,5-dinitrosalicyclic acid hydrazide (DNSH)

MDNSA (1 g) was reacted with hydrazine sulphate (0.8 g) and then suspended in 30% ammonia (200 mL). The mixture was allowed to stand for 24 h at 60 ºC and then filtered. The filtered residue was washed with ethanol and then recrystallized from methanol to give DNSH (0.7 g, 70% yield) as yellow crystals. APCI-MS analysis (negative) *m/z* was 241 [M-H]^-^ while ^1^H-NMR (600 MHz, *d*_6_-DMSO and TMS): δ 8.92 (d, *J*=3.0Hz, 1H, ArH) and 8.72 (d, *J*=3.0 Hz, 1H, ArH).

### Synthesis of 2-(2-(2-hydroxy-3,5-dinitrobenzoyl)hydrazono) acetic acid (DNSHA)

To a methanol solution (10 mL) containing glyoxalic acid (0.11 g), DNSH (0.24 g) was added. The mixture was allowed to stand for 3 h at about 25 ºC and then filtered. The filtered residue was washed three times with ethanol to give DNSHA (0.21 g, 72% yield) as a yellow powder. APCI-MS analysis (negative) *m/z* 297 [M-H]^-^; ^1^H-NMR (600 MHz, *d*_6_-DMSO and TMS): δ 14.13 (s, 1H, OH); 8.78 (d, *J*=3.0 Hz, 1H, ArH), 8.58 (d, *J*=3.0 Hz, 1H, ArH), 7.95 (s, 1H, N=CH) and 7.70 (s, 1H, NH).

### Preparation of immunizing conjugates

DNSHA (or DNSA) (10 μmol), NHS ester (10 μmol) and DCC (10 μmol) were dissolved in DMF solution (1000 μL). The mixture was gently stirred overnight at 4 ºC and then centrifuged (2,500 rpm for 10 min) to remove precipitated urea. The clear supernatant phase was collected and 900 μL of the supernatant was added to BSA or OVA (60 mg) in PBS (pH 7.4, 9 mL). The reaction mixtre was stirred overnight at 4°C and then dialyzed against 0.9% NaCl solution (1,000 mL) for 3 days at 4°C with three changes per day to give the DNSHA-BSA, DNSHA-OVA and DNSA-OVA immuno-conjugates. They were respectively diluted to 1 mg/mL with 0.9% NaCl solution and then stored at -20°C until used. The ratios of DNSHA and DNSA to carrier proteins were determined by the TNBS method.

### Preparation of polyclonal antibody to DNHS

For booster immunizations, immunogen (1 mg) was dissolved in 0.01 mol/L PBS (pH 7.4, 0.5 mL) and emulsified with Freund’s incomplete adjuvant (0.5 mL). The emulsion was then injected subcutaneously. The booster immunizations were repeated every three weeks. The New Zealand white rabbit was bled through ear vein one week after each injection. To obtain antiserum, blood samples were left to coagulate for 1 h at about 25 ºC and overnight at 4 ºC, followed by centrifugation for 10 min at 10,000 rpm. The clear supernatant phase was carefully collected. The polyclonal antibody was purified from antiserum by the ammonium sulfate precipitation method [16], then divided into aliquots and finally stored at -20 ºC until used.

### Indirect ELISA procedure

Standard DNSH and its structurally related compounds were diluted to various concentrations with PBS before the ELISA assay. The checkerboard procedure was used to optimize the coating antigen and the antibody concentrations. For the ELISA, each well of a microtitre plate was coated with 100 μL coating antigen (DNSA-OVA or DNSHA-OVA) at the optimal dilution and then incubated overnight at 4 ºC. The excess binding sites were blocked with 5% glycine for 3 h at 37 ºC. After removal from the blocking solution, the optimal antibody dilution solution (50 μL) and DNSH standard solution (50 μL) were added to the wells before the plate was incubated for 1 h at 37 ºC. After 3 washes with PBS with 0.05% Tween 20, horseradish peroxidase-labeled goat antirabbit IgG solution (100 μL) was added to each well and the plate was incubated for 20 min at 37 ºC. The plate was washed before 1% (w/v) tetramethylbenzidine (100 μL) was added. Finally, the plate was incubated for 10 min at 37 ºC before 2 mol/L H_2_SO_4_ (50 μL) was added to stop the reaction. The plate was read at 450 nm using an ELISA plate reader.

Relative absorbance was calculated using the formula B/B_0_, where B_0_ was the absorbance of the well without DNSH and B was the absorbance of the well with DNSH. The standard curves were constructed by plotting relative absorbance average values against the logarithm of DNSH concentrations with three replicates per concentration. Sigmoidal curves were fitted to four-parameter logistic equation and the standard deviations (n=3) were calculated, using Origin Program 7.5 software packages.

The specificities of the ELISAs against several veterinary chemicals were determined and calculated as the cross reaction rate (CR).


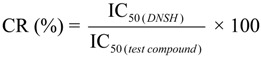


### Preparation of samples

Chicken samples were purchased in a local retail outlet. The chicken was cut into small pieces. The tested samples were respectively spiked with methanol solutions (10 μL) containing different DNSH concentrations (0.1, 0.2, 0.8 and 2.0 nmol/g chicken). A sample without DNSH was used as a negative control. The homogenized sample (1.0 g) was transferred into a glass tube and distilled water (0.9 mL) and 1 mol/L HCl (0.1 mL) were added and mixed for 10 min using a vortex mixer. After centrifugation for 2 min at 10,000 rpm, the supernatant phase was collected. The supernatant was mixed with ether (1 mL) for 60 s using a vortex mixer and then centrifuged for 2 min at 10,000 rpm. The water phase was collected after centrifugation. The DNSH content of the water phase was evaporated to dryness at 50 ºC under nitrogen. Then, methanol (2 mL) was added, mixed for 10 min using a vortex mixer and centrifuged for 2 min at 10,000 rpm. Finally, the methanol phase was evaporated to dryness at 50 ºC under nitrogen. The obtained residue was redissolved in 0.5 mL of assay buffer mentioned above and placed in an ultrasonic bath for 2 min, followed by mixing for 2 min with a vortex. DNSH content was detected by the above-mentioned indirect ELISA method.
